# Modeling Impacts of Highway Circular Curve Elements on Heavy-Duty Diesel Trucks’ CO_2_ Emissions

**DOI:** 10.3390/ijerph16142514

**Published:** 2019-07-14

**Authors:** Xiaodong Zhang, Jinliang Xu, Menghui Li, Qunshan Li, Lan Yang

**Affiliations:** 1 School of Highway, Chang’an University, Xi’an 710064, China; 2 China Harbour Engineering Company Limited, No. 9 Chunxiu Road, Dongcheng District, Beijing 100027, China; 3 Qinghai Province Traffic Construction Project Cost Management Station, Xining 810003, China; 4 Shaanxi Expressway Testing and Measuring Company Limited, Xi’an 710086, China

**Keywords:** circular curve, CO_2_ emission, heavy-duty diesel truck, MOVES model

## Abstract

Heavy-duty trucks contribute a significant component of all transportation in cargo terminals, such as Shaanxi Province, China. The emissions from these vehicles are the primary source of carbon emissions during highway operations. While several studies have attempted to address emission issues by improving traffic operations, a few focused on the relationship between emissions and highway geometric design, especially for heavy-duty trucks. The primary goal of this research was to understand the impact of circular curve on carbon dioxide (CO_2_) emissions produced by heavy-duty diesel trucks. Firstly, appropriate parameters were specified in MOVES (motor vehicle emission simulator) model according to the geometrical characteristics. Fuel consumption, speed and location data were collected by hiring five skilled drivers on the automotive proving ground located at Chang’an University, Shaanxi Province. The associated carbon emission data were derived from fuel consumption data by applying the IPCC (Intergovernmental Panel on Climate Change) method. After this, the applicability of MOVES model was verified by the field experiment. Moreover, a multiple regression model for CO_2_ emissions incorporated with roadway segment radius, circular curve length, and initial vehicle speed was established with data generated by the MOVES model. The proposed CO_2_ emission model was also verified by field experiment with relative error of 6.17%. It was found that CO_2_ emission had monotone decreasing property with radius increasing, and the minimum radius that influenced diesel CO_2_ emission was 550 m. The proposed quantitative CO_2_ emission model can provide a reference for low-carbon highway design, leading to environment-friendly transportation construction.

## 1. Introduction

Global warming caused by greenhouse gases is a severe challenge for human beings. CO_2_ is a major cause of greenhouse gas. China’s CO_2_ emissions increased significantly with rapid economic growth [1] and reducing CO_2_ emission is an arduous task in China’s efforts to tackle global climate change [2]. In China, approximately 10% of total CO_2_ emissions is emitted by the transportation sector [3], among which heavy-duty trucks account for about 20% by diesel fuel consumption. Besides, heavy-duty trucks take a large proportion of all transportation in cargo terminals, such as that in Shaanxi Province (about 39.98%) [4]. Therefore, the study of carbon emissions from heavy-duty trucks has more practical significance on these roads compared with cars.

Previous studies have indicated that fuel characteristics, traffic operations, highway horizontal and vertical alignments and vehicle engine performance can affect the vehicle’s fuel consumption and emissions [5,6,7,8,9,10,11,12,13,14,15,16,17,18,19,20,21]. Despite increasing emissions, great progress has been made in the development of engine technology and alternative fuels in recent years. GOC (the government of China) has also taken actions to relieve this situation, such as implementing environmental policies and introducing clean energy. Several models have been developed to accurately estimate emissions from cars, trucks and non-highway mobile sources under user-defined conditions, such as the MOVES (motor vehicle emission simulator) model and the STIRPAT (stochastic impacts by regression on population, affluence, and technology) model. The MOVES model has been widely recognized by scholars of various countries due to its high accuracy [6]. The United States Environmental Protection Agency (US-EPA) stated that the MOVES model was sensitive to vehicle operating modes changing [7]. STIRPAT is widely used for urban macro carbon emission scenario prediction, but it cannot be applied for microstructure prediction, while the MOVES model can achieve this goal [8]. Mathematical methods have also been used to establish fuel consumption models based on vehicle operation, such as hybrid regression model [9] and least-squares method [10]. Certain studies were carried out regarding intelligent transportation. It was reported that the speed, acceleration and power cannot effectively reflect fuel efficiency in a dynamic traffic network [11]. Obtaining data from field tests represents a heavy workload, while it is convenient to collect mass data by the MOVES model with high accuracy. Therefore, the MOVES model is applied in this research.

Carbon emission is closely related to vertical and horizontal alignment [12,13,14,15,16,17,18,19,20,21]. Most of these studies chose longitudinal slope as the research element [12,13,14,15,16]. Boriboonsomsin and Barth revealed that carbon emissions were significantly influenced by longitudinal slope utilizing the advanced navigation system [12]. Ko et al. found that the fuel consumption decreased with K (the rate of vertical curvature) increasing and increased along the vertical curves [13]. In 2013, they generated vehicle speed profiles based on grades, initial speeds and critical length of grades using the same method. Results showed that fuel consumption and emissions were reduced with faster initial speeds in the longitudinal grade and emissions from the upgraded 9% segment were about four times higher than that on a flat segment [14]. A carbon emission prediction model was established for heavy trucks incorporated with initial speed, slope, grade, and length [15]. They tried to integrate expressway longitudinal slope design with vehicle carbon emission in China [16]. It can be summarized that the initial speed is a crucial factor affecting both fuel consumption and carbon emissions. For a given longitudinal slope section, the lower initial speed may lead to higher acceleration, thus increasing the carbon emissions. However, it takes more fuel to achieve a high initial speed.

Although multiple achievements have been obtained on impacts of geometric elements on carbon emissions in endeavors of researchers, existing knowledge on horizontal alignment is still very limited. Llopis-Castelló et al. evaluated the impact of curvature change rate (CCR) and average speed on CO_2_ emission and found that the emission rate increased with CCR [17]. High CCR value means that the road segment is mainly composed of sharp curves and short tangents. Higher emissions would result from higher speed variation (i.e., acceleration, deceleration) caused by high CCR value. Nobili et al. noted that fuel consumption and CO_2_, NO_X_ and HC emission rates increased as CCR increased and average radius decreased [18]. These two studies provided a detailed analysis of the relationship between horizontal geometric design and CO_2_ emissions, but the analyses are mainly focused on the effect of CCR for passenger cars. Llopis-Castelló et al. concluded that vehicle CO_2_ emissions increased as the consistency level of a homogeneous road segment decreased [19]. The consistent road can provide the driver with a harmonious driving experience, which means that the speed is more stable with less speed variations, thus resulting in less CO_2_ emissions. In another study, the impact of road geometric design indicators on carbon emissions was studied using parameters such as longitudinal slope, radius of flat curve and vehicle speed. The study made an effort to integrate quantitative environmental evaluation into the design process [20]. On small-radius sections, the driver usually reduces the speed while cornering, which will affect fuel consumption and emissions due to speed reduction and acceleration/deceleration. From [20], it was found that fuel consumption and emissions increased with smaller radius than the minimum standard. However, only radius was chosen as horizontal alignment element in this research. The association of carbon emissions and the circular curve were evaluated for Northwestern China using radius and length of the circular curve, coupled with the initial velocity as the variables. It was found that the minimum curve radius impacting carbon emissions was 500 m for a 30 ton load heavy-duty truck reported only on the basis of field test data which was limited [21]. From the previous studies [18,20,21], it can be concluded, on circular curve, carbon emission is influenced by both radius and curve length. Other horizontal geometric elements such as CCR have certain influence on driving operation and CCR has been studied by many scholars [17,18,19], and radius was primarily chosen as the research variable in this paper, but that is not in the scope of this research. In collecting data, most scholars preferred model simulation [17,18,19,20].

The existing studies on horizontal alignment mainly examined carbon emissions from passenger cars [17,18,19]. Moreover, the road elements are not comprehensively studied [17,18,19,20]. Compared with passenger cars, HGVs (heavy goods vehicles) are influenced greatly with small-radius curve sections [22]. The transverse force on HGVs is greater with high gravity center. Influenced by transverse force, HGVs need to overcome the friction between tires and road surface while cornering, increasing fuel penalty. In understanding the impact of horizontal alignment on carbon emissions from heavy-duty trucks, this research analyzed the relationship between horizontal alignment elements (radius, circular curve length and initial speed) and cumulative CO_2_ emission. By applying the MOVES model, large number of energy consumption and truck operation data were obtained. Then a novel CO_2_ emission prediction model was proposed. The applicability of the proposed model was further validated using field experiment data. The advantages of the proposed model are two-fold. Firstly, the model can precisely predict the cumulative CO_2_ emission from heavy trucks. Secondly, the three variables, circular curve radius, curve length and initial speed are comprehensively considered in model construction, filling the research gap on impacting of horizontal alignment on CO_2_ emission. The findings will be promising in environmentally-friendly highway design and sustainable transportation construction.

The rest of the paper is organized as follows. The fundamentals of the MOVES model are presented in Section 2. Section 3 describes the evaluation of heavy-duty diesel truck fuel consumption and emission. The modeling and validation of the CO_2_ emission prediction model is also outlined. The whole research is discussed in Section 4 and summarized in Section 5.

## 2. Methods and Data

There are many alignment combinations for highway horizontal alignment. It is difficult to obtain data under combinations of different radii and curve lengths only by field experiment because on-site alignments are heavy workloads, but the MOVES model can easily achieve this goal. In this section, parameters in the MOVES model are well chosen and then are verified by a field experiment carried out in Chang’an University, Shanxi Province. AIC (Akaike’s information criterion) and BIC (Bayesian information criterion) criteria are employed to evaluate the goodness-of-fit for modeling.

### 2.1. Motor Vehicle Emission Simulator (MOVES) Model Parameters Selection

In the modeling process, the user specifies time period, geographical area, vehicle type, road type, fuel type and vehicle operating characteristics to be modeled. The model then performs a series of calculations, which have been carefully developed to accurately reflect vehicle operating processes. In this study, model parameters were determined according to the basic conditions of Xi’an city, Shaanxi Province.

(1) Time period: multiple differences exist in diesel emission standards and diesel quality between China and the United States. At present, diesel oil adopted in Shaanxi province is regulated by stage-V emission standard of China. Therefore, the simulation time period was determined to be the year 2000 after matching the relevant parameters of the United States and China. Detailed comparison of diesel quality between the two nations is shown in Table 1 [23].

(2) Geographical area: the field experiment was completed at Chang’an University automotive proving ground in Xi’an City, Shaanxi Province. The similarity between Xi’an and Missouri State is noticeable compared with geographic and climatic conditions in other states. Therefore, Missouri state was selected as the simulated geographical area. Detailed comparison of geographic features of Xi’an city and Missouri are shown in Table 2.

(3) Vehicle type: a Jiefang Wellway J5M heavy-duty diesel truck (CA2120P7K2T5A70E3, weight 12 ton) was chosen as the test vehicle. Referring to the vehicle types in the MOVES model, the corresponding vehicle type was identified as single-unit long-haul truck with ID 53 (Table 3).

(4) Road type: this paper mainly focused on arterial highways and highways with design speeds greater than 60 km/h and non-restrictive types. From the MOVES database, the road type chosen to model was ID 5 (urban non-restrictive road).

(5) Fuel type: referring to the vehicle diesel standard (GB19147-2016) and the relevant parameters of the Chinese diesel quality presented in Table 1, the diesel type was determined as diesel oil with code 20043.

When using the MOVES model, it is essential to calculate the VSP (vehicle specific power) according to the instantaneous speed and acceleration. On this basis, the operating mode value (OMV) was estimated by dividing the highway into different sections. Therefore, the accuracy of the simulation results is partially determined by the selection of instantaneous acceleration and instantaneous speed models. In this research, it was assumed that drivers applied a constant deceleration prior to the curve midpoint and a constant acceleration before they left the curve, and the matching instantaneous speed model in MOVES used in this paper is shown in Equation (1) [24,25]:

(1) $$\{\begin{array}{c} V\left(t\right)=V_{0}+\alpha_{1}t\;\left(0\leq t\leq t_{1}\right)\\ V\left(t\right)=V_{m}+\alpha_{2}t\;\left(t_{1}\leq t\leq t_{2}\right) \end{array}$$

where $V_{0}$ is the initial speed (km/h); $\alpha_{1}$ is the deceleration from the beginning to the midpoint of the curve (m/$s^{2}$), the calculation equation is shown in Table 4; $t_{1}$ is the total driving time from the starting point to the midpoint of circular curve (s), $t_{1}=\frac{V_{m}-V_{0}}{\alpha_{1}}$; $V_{m}$ is the speed at the midpoint of the circular curve (km/h), $V_{m}=\sqrt{V_{0}^{2}+2\alpha_{1}s_{1}}$, $s_{1}=\frac{S}{2}$, S is the total length of the circular curve (m); $\alpha_{2}$ is the acceleration from midpoint to endpoint of circular curve (m/$s^{2}$) and is shown in Table 5; $t_{2}$ is the total driving time from the midpoint to the endpoint of the circular curve (s), $t_{2}=\frac{V_{f}-V_{m}}{\alpha_{2}}$, $V_{f}$ is the speed at the endpoint of the circular curve (km/h), $V_{f}=\sqrt{V_{m}^{2}+2\alpha_{2}s_{2}}$; $s_{2}=\frac{S}{2}$.

### 2.2. MOVES Model Verification

Since the parameters in MOVES are obtained according to vehicle emission data in United States of America, thus the applicability in China needs to be tested. A field experiment was designed to reach this goal.

**Figure 1:**
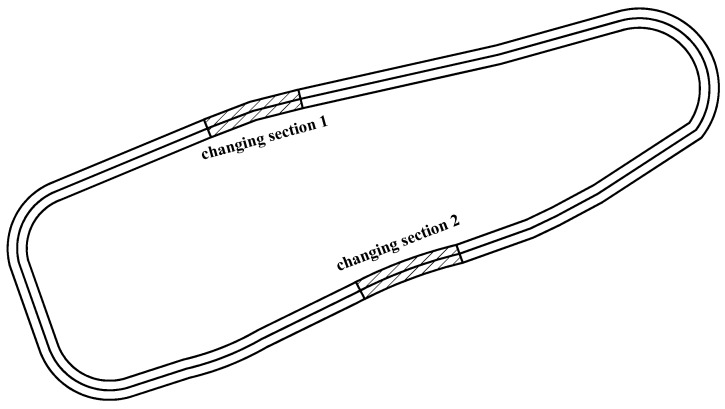
Horizontal alignment of experimental road.

An on-site alignment was designed at the automobile test ground in Chang’an University to eliminate the impacts of environmental factors on vehicle performance, thus achieving free-flow operation. The geometric features of the experimental road were consistent except for the horizontal curve, and no vertical curve was set. This paper primarily focused on arterial highways and highways with design speeds greater than 60 km/h. The Chinese Design Specification for Highway Alignment regulates a general value of minimum radius 200 m for design speeds larger than 60 km/h. In addition, previous studies have shown that the influence of radius on the running speed was not obvious when the radius was larger than 550 m [24], and the maximum circular radius of the field experiment was tentatively set at 550 m, which will be tested by the following study. Based on the above analysis, the range of radius chosen in this paper was [200 m, 550 m]. Compared with cars, heavy trucks are influenced greatly on small-radius curve section [22]. For sections with curve radius greater than 200 m, the setting of transition curve has no obvious impact on road traffic safety and in this case, the setting of the transition curve has little effect on driver’s driving behavior. Thus, the effect of transition curve on carbon emissions can be neglected in this research [26]. By changing the radius of changing Section 1 and Section 2 (Figure 1), the CO_2_ emission level of diesel trucks with different radii and lengths were measured. The radii of the two sections are shown in Table 6. The fuel consumption data on straight sections were also recorded to determine the minimum curve radius affecting CO_2_ emission later. The impact of a truck’s weight on carbon emissions was not considered in this research, so a Jiefang Wellway J5M heavy-duty diesel truck (CA2120P7K2T5A70E3) with total weight of 12 tons was chose as the typical diesel truck [4]. 5 experienced truck drivers (male, aged 35 to 39 years, with driving experience of 10 to 18 years) were employed in field experiment. Before the test, they were requested to join the training to be familiar with the test road, thus maximumly eliminating the driver behavioral impacts on the results. A JDSZ-EP-1-1D Fuel Consumption Meter was used for fuel consumption measurement. Instantaneous speed, fuel consumption and cumulative fuel consumption data were dynamically measured and recorded by this instrument.

The experiment was carried out from 24 May to 29 May 2018 at the automotive proving ground under clear weather. The test vehicle and fuel consumption meter were checked before the test. The drivers took turns repeating 56 cycles along the designated radius, and 56-cycle for one driver was one group of data for a designated radius. The repeatability of the operation could maximumly eliminate uncertain factors during the experiment. Detailed fuel consumptions, initial speeds, final speeds and locations were all recorded. A total of 40 groups of data with all designated radii were collected.

Fuel consumption data were obtained from the field experiment by the fuel consumption meter. The IPCC (Intergovernmental Panel on Climate Change) accounting method was applied to convert fuel consumption data into carbon emission data. The IPCC accounting method is internationally recognized and widely used in estimation of greenhouse gas emissions [27]. The detailed description of the method is explained by Equations (2)–(5).

(2) CEF=NCV×PF×COF×K

(3) $${\mathrm{CEFL}}_{i}=\mathrm{CEF}\times \rho_{i}$$

(4) $$E_{i}={\mathrm{CEFL}}_{i}\times \Delta T_{i}$$

(5) $$e_{i}=E_{i}/S_{i}$$

where CEF is CO_2_ emission coefficient (kg CO_2_/kg); NCV is average net calorific value (TJ/Gg); PF is the potential carbon emission coefficient (t-C/TJ); COF is carbon oxidation ratio (%); K is efficiency of carbon conversion which is equal to 44/12; ${\mathrm{CEFL}}_{i}$ is CO_2_ emission coefficient for different diesel oil types per liter; $\rho_{i}$ is the density of different diesel oils (${\mathrm{kg}/m}^{3}$); $\Delta T_{i}$ is the cumulative fuel consumption of 56-cycle (kg); $E_{i}$ is the total CO_2_ emission (kg); $e_{i}$ is the carbon emission per unit length (kg/m); $S_{i}$ is the total length of 56-cycle (m).

In the MOVES model, energy consumption was firstly collected and then converted to fuel consumption as shown in Equation (6). Fuel consumption was converted to carbon emission using the same method mentioned in Equations (2)–(5).

(6) Fuel consumption (kg)=energy consumption (kg)/29,270×1.4571

The precision of the instantaneous speed model was verified by the field test data with different radii. The predicted speed and the experimental speed are shown in Table 7. The speed difference between the predicted speed and the experimental speed was calculated to verify whether the selected parameters met the accuracy requirement.

As shown in Table 7, the relative error between the predicted value and the experimental value with different radii ranged from 1.66% to 3%, which verifies the selected speed model can meet the accuracy requirements and can be used in MOVES.

By inputting continuous parameters as mentioned in 2.1, CO_2_ emission data were obtained. The precision of the MOVES model in China was evaluated by the difference between the simulated and actual CO_2_ emission.

As shown in Table 8, the average relative error is 7.34%. Therefore, the parameters selected in the MOVES model can meet the accuracy requirements, and MOVES model can be used to simulate CO_2_ emission with different radii and lengths in China.

### 2.3. Model Criterion

In the following modeling process, both AIC and BIC criteria were employed to evaluate the goodness-of-fit for the regression models. The accuracy of the model is higher when the values of AIC and BIC are relatively small, that the interpretation of data is better.

## 3. Results

### 3.1. CO_2_ Emission Database

**Figure 2:**
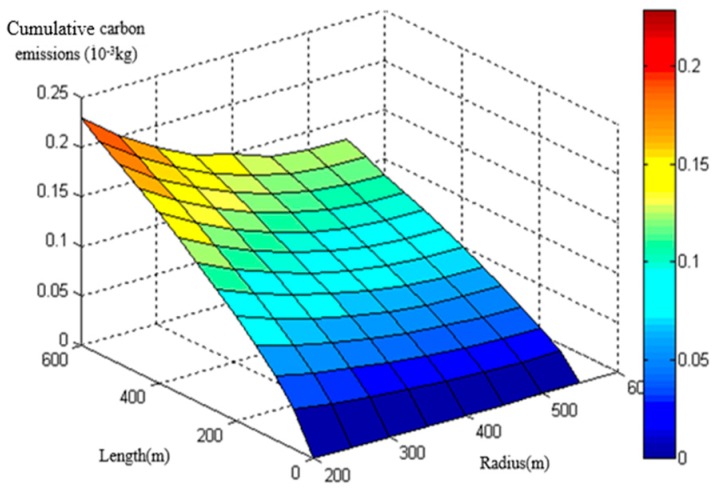
CO_2_ emission database of heavy-duty diesel trucks at an initial speed of 30 km/h.

**Figure 3:**
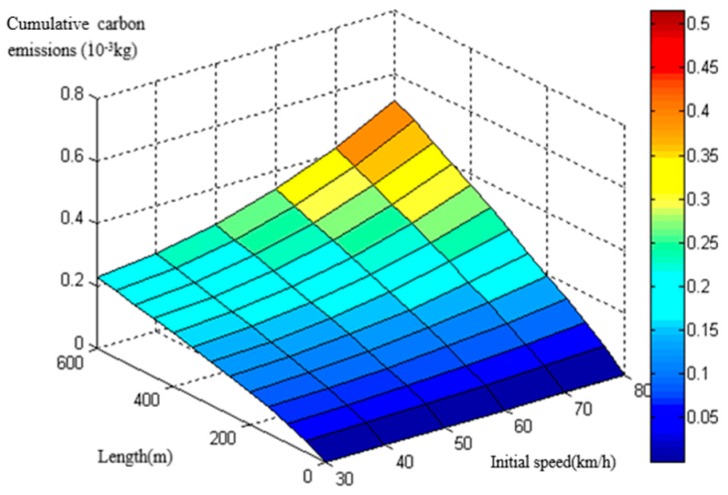
CO_2_ emission database of heavy-duty diesel trucks with a radius of 200 m.

The basic modeling data derived from the MOVES model. By inputting continuous parameters as mentioned in 2.1, a considerable amount of CO_2_ emission data were obtained. The database of heavy-duty diesel trucks on the circular curve section was then established through the MOVES model (Figure 2 and Figure 3). The following models were built on the basis of the database.

### 3.2. CO_2_ Emission Prediction Model

#### 3.2.1. Unary Regression Model of CO_2_ Emissions

**Figure 4:**
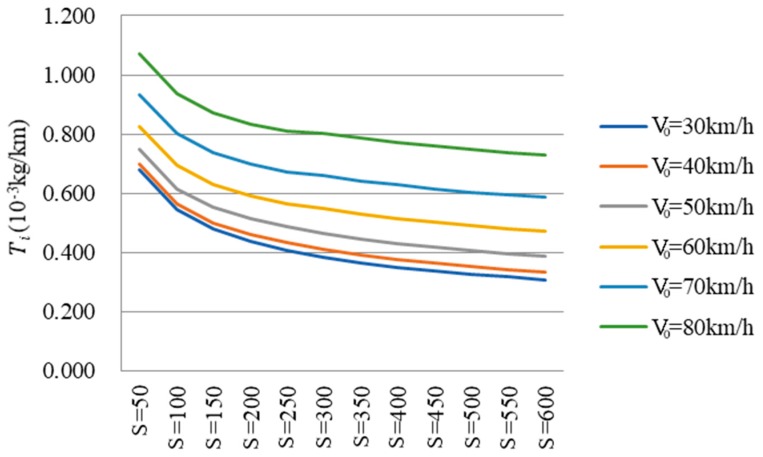
$T_{i}$ with a radius of 200 m.

**Figure 5:**
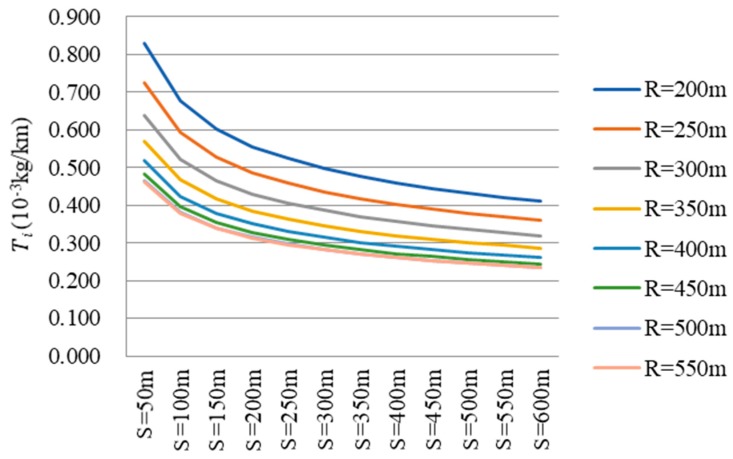
$T_{i}$ with an initial speed of 40 km/h.

In the modeling process, CO_2_ emissions per unit length $T_{i}$ was chosen as the dependent variable because it is more concise than cumulative CO_2_ emissions *T*. $T_{i}$ is the quotient of *T* and *S*, and the results were then converted to cumulative CO_2_ emissions. As shown in Figure 4 and Figure 5, $T_{i}$ displayed an obvious trend on circular curve sections. Therefore, it is feasible to model between $T_{i}$ and the three variables (radius, length and initial speed) and SPSS software was used to fit the relationships. The unary regression models were firstly built and then a multiple regression model could be proposed.

**Figure 6:**
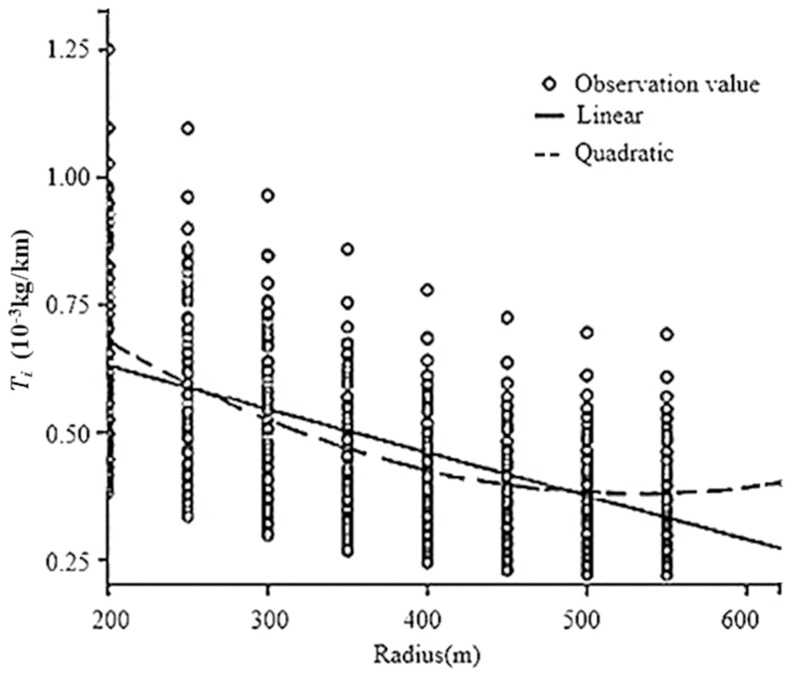
Regression models.

The unary regression modeling process was demonstrated by taking the radius as an example. The fitting results are shown as Figure 6, Table 9 and Table 10.

As presented in Table 9 and Table 10, the AIC and BIC values of the quadratic model were relatively small with R^2^ = 0.736, significance, Sig. = 0.000. Overall, the quadratic regression line showed the best fit.

Based on the above analysis, the best regression model between CO_2_ emissions per unit length and the radius was expressed as Equation (7):

(7) $$f\left(R\right)=1.150-0.003R+2.734{\times 10}^{-6}R^{2}$$

The same process was carried out to analyze the other two parameters. The best three unary regression models are listed in Table 11.

#### 3.2.2. Multiple Regression Model

A multiple regression model was proposed (Equation (9)) based on the unary regression models, to comprehensively reflect the influence of the initial speed, circular curve length and radius on CO_2_ emissions through SPSS. The regression results are shown in Table 12 and Table 13.

The regression model for $T_{i}$ is:

(8) $$T_{i}=-0.866+0.912f_{1}\;\left(R_{i}\right)+1.005f_{2}\left(S_{i}\right)+0.996\;f_{3}\;\left(V_{0i}\right)$$

Finally, the regression models for T is:

(9) $$T=\overset{n}{\underset{i=1}{\sum }}(S_{i}\cdot (0.618-0.002736\cdot R_{i}+2.493{\times 10}^{-6}\cdot R_{i}^{2}+1.56378\cdot S_{i}^{-0.223}-0.006149\cdot V_{0i}+0.000116\cdot V_{0i}^{2}))$$

where *T* is the cumulative CO_2_ emissions (10^−3^ kg); $S_{i}$ is the *i*th circular curve length (m); $R_{i}$ is *i*th circular radius (m); $V_{0i}$ is the *i*th initial speed (km/h).

It is important to note that Equation (9) can only be used to calculate the cumulative CO_2_ emissions when the radius in the range of [200 m, 550 m].

#### 3.2.3. Model Validation

The accuracy of the proposed CO_2_ emission prediction model was validated using field experiment data. During the validation process, only 1-cycle length of the experimental road was used to calculate CO_2_ emission for both the prediction model and the experiment. From Table 14, the average relative error between predicted and actual CO_2_ emission is 6.17%, which indicated high accuracy of the proposed prediction model, and the applicability of the CO_2_ emission prediction model in China is guaranteed.

### 3.3. Minimum Curve Radius Affecting CO_2_ Emissions

**Figure 7:**
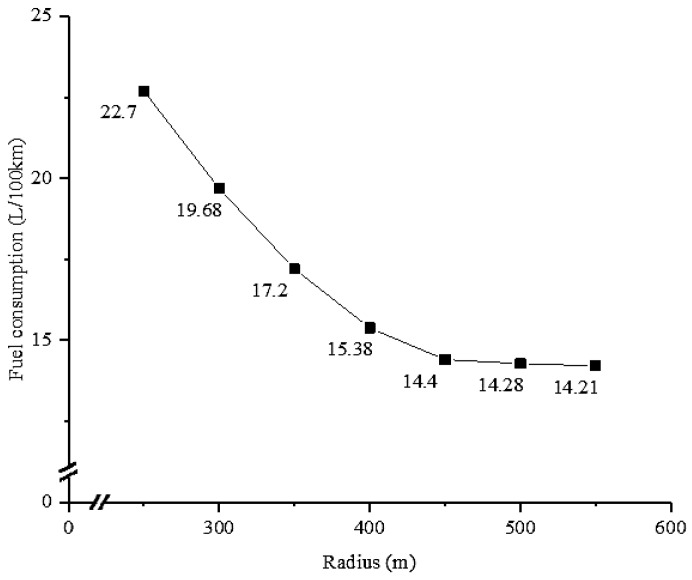
Fuel consumption with different radii.

One of the hypotheses in this paper is that the impact of radius on carbon emissions is minimal when the radius is larger than 550 m. The fuel consumption data from field experiment is shown in Figure 7. It is apparent that higher radii result in lower fuel consumption. The slope is practically zero when the radius is 550 m. The reduction in fuel consumption is as small as 0.07 L/100 km when the radius changes from 500 m to 550 m. The fuel consumption for 550 m circular curve is 14.21 L/100 km, which is close to that of straight section (14.09 L/100 km). Therefore, the finding is in agreement with the initial assumption, and 550 m can be determined as the minimum radius affecting CO_2_ emission.

## 4. Discussion

The research simulated the actual conditions of Xi’an city by matching reasonable parameters of the MOVES model. Based on the MOVES model, the fuel consumption data under different radii were obtained. The CO_2_ emission data were transformed from the fuel consumption data by applying IPCC accounting method. Finally, a CO_2_ emission prediction model was proposed incorporated with radius, circular curve length and initial speed. The accuracy of the model was validated by the field experiment data. The applicability of the prediction model in China is guaranteed with the relative error of 6.17%.

The minimum circular radius affecting CO_2_ emissions is 550 m from this research. However, this finding does not agree with the finding from [21], where the minimum curve radius impacting carbon emissions was found to be 500 m. The difference in results from this study and reported literature might be attributed to the differences in a heavy-duty truck’s weights (30 tons used in the prior study and 12 tons in this study). Therefore, it might be plausible to speculate that the minimum curve radius is negatively correlative with the truck weight and this can be further explored. The relationships between geometric indexes and carbon emissions are qualitatively analyzed in [17,18,19,20], while a CO_2_ emission prediction model comprehensively considering radius, curve length and initial speed is proposed in this paper, which can be used for quantitative analysis. The impact of average radius on fuel consumption and carbon emission are studied in [18], revealing fuel consumption and CO_2_ emission rates increase as average speeds decrease. The radius in [20] is below the minimum radius standard and the research show that fuel consumption and emissions increase with smaller radius. In this paper, the radius is in [200 m, 550 m], and the findings reveal that CO_2_ emission decreases with the increase of radius, and when the radius is larger than 550 m, the increase of radius has little impact on carbon emission. In this research, the radius was restricted to [200 m, 550 m] and the influence of the transition curve was ignored. Meanwhile, a typical heavy-duty truck was selected as a test vehicle, so the CO_2_ emission model cannot predict the carbon emissions of other vehicles. In future, the characteristics of carbon emissions with radius smaller than 200 m can be investigated. Moreover, the prospective study of carbon emission can take other factors (e.g., transition curve, vehicle mass, tyres and axles) into consideration. The carbon emissions under the combination of horizontal and vertical alignments can also be further studied.

## 5. Conclusions

This research attempted to study the relationship between CO_2_ emissions and highway horizontal geometric elements for heavy-duty diesel trucks, thus guiding environmentally conscious highway construction. The present research provided a novel CO_2_ emission prediction model for heavy-duty truck’s CO_2_ emissions on circular curves and identified the minimum curve radius affecting CO_2_ emissions.

The proposed CO_2_ emission prediction model can be widely used to analyze the potential CO_2_ emissions during the design and long-term usage of the highway. Moreover, the cumulative CO_2_ emission can be evaluated as an environment index in scheme comparison and selection. The result of the minimum curve radius affecting the carbon emissions has the potential to offer a reference for low-carbon highway design of horizontal curves.

## Figures and Tables

**Table 1:** Summary of diesel quality in the United States and China.

Parameters	Sulfur Content	T90 °C	T95 °C	Viscosity	Cetane	Density
	(≯ppm)	(≤)	(≤)	(mm^2^/s)	Number (≯)	(kg/m^3^)
China (2018)	50	355	365	3.89	47–51	790–840
United States (2000)	50	310	320	4.0	42	840

**Table 2:** Geographic features in Xi’an and Missouri.

Parameters	Xi’an	Missouri State
Latitude	33°39′ N–34°45′ N	36° N–40°35′ N
Longitude	107°40′ E–109°49′ E	89°6′ W–95°42′ W
Altitude	400 m	240 m
Annual average temperature	13.1 °C–13.4 °C	13 °C
Annual average precipitation	500–750 mm	693 mm
Relative humidity	58%	62.5%

**Table 3:** Vehicle type in test and model.

Parameters	Jiefang Wellway J5M	Single-Unit Truck
Number of axles	2	2
Number of tyres	6	≥6
Gross vehicle weight rating	12 tons	≥10,000 lb (4.536 tons)

**Table 4:** Equation for calculating deceleration from starting point to midpoint of circular curve.

Radius (m)	$\mathrm{Deceleration}\;(m/s^{2})$
R ≤ 175	−1
175 < R≤ 476	$\alpha_{1}=-0.6794-\frac{295.14}{R}$
476 ≤ R < 575	$\alpha_{1}=-0.2794-\frac{295.14}{R}$
R > 575	0

**Table 5:** Acceleration from midpoint to endpoint of circular curve.

Radius(m)	$\mathrm{Acceleration}\;(m/s^{2})$
175 < R ≤ 250	0.54
250 < R ≤ 476	0.43
476 < R ≤ 875	0.21
R > 875	0

**Table 6:** Radius of changing sections.

Number	Radius of Changing Section 1 (m)	Radius of Changing Section 2 (m)
1	200	200
2	250	250
3	300	300
4	350	350
5	400	400
6	450	450
7	500	500
8	550	500

**Table 7:** Comparison of predicted and experimental speed.

No.	Radius	Length	Predicted Value	Experimental Value	Speed Difference	Relative Error
	(m)	(m)	(km/h)	(km/h)	(km/h)	(%)
1	250	45.6	52.708	53.664	−0.956	1.78
2	300	54.7	48.013	48.570	−0.558	1.15
3	350	63.8	43.315	43.479	−0.164	0.38
4	400	72.9	38.620	39.141	−0.521	1.33
5	450	82.0	33.923	34.828	−0.905	2.60
6	500	91.2	32.506	31.888	0.618	1.94
7	550	100.3	33.638	32.832	0.806	2.46

**Table 8:** Comparison of simulated and actual CO_2_ emissions.

No.	Radius	Length	Predicted Value	Actual Value	CO_2_ Emission Difference	Relative Error
	(m)	(m)	(kg)	(kg)	(kg)	(%)
1	250	45.6	0.029	0.027	0.002	6.06
2	250	36.5	0.023	0.022	0.001	3.70
3	300	54.7	0.031	0.029	0.002	5.71
4	300	54.0	0.030	0.028	0.002	5.88
5	350	63.8	0.031	0.029	0.002	8.57
6	350	63.0	0.031	0.029	0.002	8.57
7	400	72.9	0.032	0.030	0.002	8.33
8	400	72.0	0.032	0.030	0.002	8.33
9	450	82.0	0.035	0.031	0.003	10.53
10	450	81.0	0.034	0.031	0.002	7.89
11	500	91.2	0.037	0.035	0.002	7.14
12	500	89.9	0.036	0.034	0.002	7.32
13	500	89.9	0.029	0.027	0.002	6.06
14	550	100.3	0.023	0.022	0.001	3.70

**Table 9:** Fitting parameters.

Regression	R^2^	F	df1	df2	Sig.	Constant	b1	b2	b3
Linear	0.705	251.317	1	574	0.000	0.801	−0.001	—	—
Quadratic	0.736	144.850	2	573	0.000	1.150	−0.003	2.734 × 10^−6^	—

**Table 10:** Residuals, Akaike’s information criterion (AIC) and Bayesian information criterion (BIC) values.

Regression	Residuals	AIC	BIC
Linear	8.558	1240.5951	1249.3073
Quadratic	7.993	1205.4112	1214.1304

**Table 11:** Unary regression models.

Model	Description	R^2^	F
$f\left(R\right)=1.150-0.003R+2.734{\times 10}^{-6}R^{2}$	Quadratic	0.736	144.850
$f\left(S\right)=1.566S^{-0.223}$	Power	0.738	151.041
$f\left(V_{0}\right)=0.437-0.006174V_{0}+0.000116V_{0}^{2}$	Quadratic	0.727	213.564

**Table 12:** Model summary.

Model	R	R^2^	Adjusted R^2^	Durbin-Watson
Multiple regression	0.983a	0.967	0.967	0.362

**Table 13:** Coefficient.

Model	Non-Standardized Coefficient B	Standard Error of Non-Standardized Coefficient	T	Significance
Constant	−0.866	0.011	−78.789	0.000
$f_{1}\left(R\right)$	0.912	0.012	76.377	0.000
$f_{2}\left(S\right)$	1.005	0.017	59.619	0.000
$f_{3}\left(V_{0}\right)$	0.996	0.012	86.165	0.000

**Table 14:** Relative errors of predicted and experimental CO_2_ emissions.

No.	Radius	Length	Initial Speed	Predicted Value	Experimental Value	Prediction
	(m)	(m)	(km/h)	(10^−3^ kg/km)	(10^−3^ kg/km)	Error (%)
1	250	45.6	36.175	686.33	723.68	5.16
2	250	36.5	36.088	720.09	739.73	2.65
3	300	54.7	36.894	593.23	639.85	7.29
4	300	54.0	36.758	594.75	629.63	5.54
5	350	63.8	37.602	517.61	548.59	5.65
6	350	63.0	37.417	518.88	555.56	6.60
7	400	72.9	37.857	456.82	493.83	7.49
8	400	72.0	37.922	458.66	486.11	5.65
9	450	82.0	38.251	411.47	439.02	6.28
10	450	81.0	38.101	412.67	444.44	7.15
11	500	91.2	38.380	379.73	405.70	6.40
12	500	89.9	38.125	380.87	411.57	7.46
13	500	89.9	38.271	381.26	417.13	8.60
14	550	100.3	38.426	361.94	378.86	4.47
